# Enantiomeric Separation and Degradation of Benoxacor Enantiomers in Horticultural Soil by Normal-Phase and Reversed-Phase High Performance Liquid Chromatography

**DOI:** 10.3390/ijms24108887

**Published:** 2023-05-17

**Authors:** Haoxiang Zhu, Kunrong Qin, Ping Zhang, Haiyang Wang

**Affiliations:** 1 College of Horticulture and Landscape Architecture, Southwest University, Chongqing 400715, China; zhuhx8910@swu.edu.cn (H.Z.); zpcauz@163.com (K.Q.); 2 College of Plant Protection, Southwest University, Chongqing 400715, China

**Keywords:** benoxacor, chiral separation, enantiomers, enantioselectivity, soil degradation

## Abstract

The separation of benoxacor enantiomers on six commercial chiral columns was investigated by high-performance liquid chromatography (HPLC) under normal-phase and reversed-phase conditions. The mobile phases included hexane/ethanol, hexane/isopropanol, acetonitrile/water, and methanol/water. The effects of the chiral stationary phases (CSPs), temperature, and mobile phase composition and ratio on the separation of benoxacor enantiomers were examined. Under normal-phase conditions, the two benoxacor enantiomers were completely separated on Chiralpak AD, Chiralpak IC, Lux Cellulose-1, and Lux Cellulose-3 columns and partially separated on a Lux Cellulose-2 column. Under reversed-phase conditions, benoxacor enantiomers were completely separated on a Lux Cellulose-3 column and partially separated on Chiralpak IC and Lux Cellulose-1 columns. Normal-phase HPLC performed better than reversed-phase HPLC for the separation of benoxacor enantiomers. As the column temperature increased from 10 °C to 4 °C, the enthalpy (ΔH) and entropy (ΔS) results indicated that the resolution was strongly affected by the temperature and that the lowest temperature did not always produce the best resolution. An optimized separation method on the Lux Cellulose-3 column was used to investigate the stability of benoxacor enantiomers in solvents and the degradation of benoxacor enantiomers in three types of horticultural soil. Benoxacor enantiomers were stable, and degradation or racemization were not observed in methanol, ethanol, isopropanol, acetonitrile, hexane, or water (pH = 4.0, 7.0, and 9.0). In three horticultural soils, the degradation rate of S-benoxacor was faster than that of R-benoxacor, resulting in soil enrichment with R-benoxacor. The results of this study will help to improve the risk assessment of enantiomer levels of benoxacor in the environment.

## 1. Introduction

Benoxacor ((RS)-4-(dichloroacetyl)-3,4-dihydro-3-methyl-2H-1,4-benzoxazine; CAS: 98730-04-2; Figure 1) is a chiral herbicide safener for S-metolachlor that is used to increase the tolerance of maize to S-metolachlor under both normal and adverse environmental conditions [1,2]. According to field data, over two million kilograms of dichloroacetamide safeners, including benoxacor, have been used in the United States, which exceeds the total amount of many active herbicides [3]. Commercial benoxacor contains two enantiomers, R-benoxacor and S-benoxacor, at a ratio of 1:1. The enantiomers of chiral pesticides generally have different or opposite biological behaviors because of enantio-specific interactions with biological molecules [4,5,6,7,8,9,10,11,12]. Enantiomer-specific toxicity, degradation, accumulation, and metabolism have become a hot spot of research in agrochemical studies in recent years [13,14,15]. Thus, it is essential to study the biological enantio-selectivity of chiral pesticides at the enantiomer level in order to commercialize highly active and environmentally friendly enantiomer pesticides [16,17]. 

In enantiomer-specific studies, separating the enantiomers of a chiral pesticide is the first and fundamental step. Gas chromatography (GC) [18], high-performance liquid chromatography (HPLC) [19,20], capillary electrophoresis (CE) [21], cyclodextrin-modified micellar electrokinetic chromatography (CD-MEKC) [22], supercritical fluid chromatography (SFC) [23], and ultraperformance convergence chromatography (UPCC) [24] are widely used to separate enantiomers. To date, HPLC coupled with various chiral stationary phases (CSPs) is the most efficient method for chiral separation. Polysaccharide-based chiral stationary phases, including cellulose-tris-(4-methylbenzoate), cellulose-tris-(4-chloro-3-methylphenylcarbamate), amylose-tris-(3,5-dimethylphenylcarbamate), and cellulose-tris-(3-chloro-4-methylphenylcarbamate) are the most commonly used CSPs because of their excellent ability to recognize chiral compound enantiomers [25,26,27,28]. As for benoxacor, few studies have examined the chiral separation of benoxacor enantiomers. Furthermore, no study has reported the benoxacor enantiomer stability or degradation at the enantiomer level.

In this study, separations of benoxacor enantiomers on six commercial chiral columns were investigated by HPLC under normal-phase and reversed-phase conditions. The effects of CSPs, temperature, and the mobile phase composition and ratio on the separation of benoxacor enantiomers were investigated. The stability of benoxacor enantiomers in different solvents and the degradation of benoxacor enantiomers in three horticultural soils were also investigated. The S-benoxacor degraded faster than R-benoxacor in the three horticultural soils, and the enantioselective degradation was highly related to the soil pH and microbial activity. The results of this study will help to improve the risk assessment of enantiomer levels of benoxacor in the environment.

## 2. Results and Discussion

### 2.1. Benoxacor Enantiomer Separation with Normal-Phase HPLC

Benoxacor enantiomers were separated on six different chiral columns using hexane/isopropanol (HEX/IPA) and hexane/ethanol (HEX/ETOH) as the mobile phase under the normal-phase HPLC condition. Results for the mobile phase composition, mobile phase ratio, separation factor (α), retention factors of the two benoxacor enantiomers (k_1_, k_2_), and resolution factor (R_s_) are summarized in Table 1. Baseline separation of the two benoxacor enantiomers was defined as R_s_ > 1.50. When the mobile phase was HEX/IPA, benoxacor enantiomers were baseline separated on Chiralpak IC and Lux Cellulose-3 columns and partially separated on Chiralpak AD and Lux Cellulose-1 columns. The maximum R_s_ values were 3.45 on Chiralpak IC (98/2), 4.15 on Lux Cellulose-3 (95/5), 1.14 on Chiralpak AD (98/2), and 0.71 Lux Cellulose-1 (75/25). Thus, for the benoxacor enantiomers, the best performance was by Lux Cellulose-3 when HEX/IPA was used as the mobile phase. When the mobile phase was HEX/ETOH, benoxacor enantiomers were completely separated on the Chiralpak AD, Chiralpak IC, Lux Cellulose-1, and Lux Cellulose-3 columns and partially separated on the Lux Cellulose-2 column. The maximum R_s_ values were 3.52 on Chiralpak AD (98/2), 2.97 on Chiralpak IC (98/2), 2.57 on Lux Cellulose-1 (98/2), 4.69 on Lux Cellulose-3 (95/5), and 0.71 on Lux Cellulose-2 (95/5). Thus, Lux Cellulose-3 also performed the best when HEX/ETOH was used as the mobile phase. To summarize, under the normal-phase condition, the best separation performance was by the Lux Cellulose-3 column, regardless of whether HEX/IPA or HEX/ETOH was the mobile phase. In addition, the Chiralpak IC and Lux Cellulose-3 columns had better separation capability than the Chiralpak AD, Lux Cellulose-1, and Lux Cellulose-2 columns, which indicates that the cellulose-tris-(3,5-dichlophenylcarbamate) in Chiralpak IC and the cellulose-tris-(4-methylbenzoate) in Lux Cellulose-3 have superior chiral recognition for benoxacor enantiomers under the normal-phase HPLC condition. Furthermore, HEX/ETOH had a better (Chiralpak AD, Lux Cellulose-1, Lux Cellulose-3, Lux Cellulose-2) or the same (Chiralpak IC) separation ability under the normal-phase condition, which indicates that interactions on the same chiral stationary phase differ between the HEX/ETOH and HEX/IPA systems. Under normal-phase HPLC conditions, compared with a relatively high alcohol content, a relatively low alcohol content generally resulted in an increased retention time and improved separation resolution for enantiomers. The decreases in the k_1_, k_2_, and R_s_ values were consistent with this phenomenon when the proportion of IPA or ETOH in the mobile phase increased.

### 2.2. Benoxacor Enantiomer Separation with Reversed-Phase HPLC

When the mobile phase was methanol/water (MEOH/H_2_O) in reversed-phase HPLC, benoxacor enantiomers were baseline separated on a Lux Cellulose-3 column and partially separated on Chiralpak IC and Lux Cellulose-1 columns. Chiralpak AD, Lux Cellulose-2, and Lux Cellulose-4 did not separate benoxacor enantiomers using MEOH/H_2_O as the mobile phase. The best resolution factors were 6.08 on Lux Cellulose-3 (80/20), 0.96 on Chiralpak IC (70/30), and 0.54 on Lux Cellulose-1 (100/0). Thus, a Lux Cellulose-3 column had the best performance when MEOH/H_2_O was used as the mobile phase. When the mobile phase was acetonitrile/water (ACN/H_2_O), benoxacor enantiomers were baseline separated only on a Lux Cellulose-3 column, with a maximum R_s_ of 5.17 at an ACN/H_2_O ratio of 50/50. Chiralpak AD, Chiralpak IC, Lux Cellulose-1, Lux Cellulose-2, and Lux Cellulose-4 columns did not separate benoxacor enantiomers when the mobile phase was ACN/H_2_O. Overall, the Lux Cellulose-3 column was the best for benoxacor enantiomer separation whether MEOH/H_2_O or ACN/H_2_O was used as the mobile phase, which indicated that cellulose-tris-(4-methylbenzoate) in Lux Cellulose-3 had superior chiral recognition of benoxacor enantiomers under the reversed-phase HPLC condition. According to the results on the Chiralpak IC and Lux Cellulose-1 columns, separation varied even on the same chiral column. In a comparison of mobile phase compositions, MEOH was shown to be both a hydrogen donor and acceptor, whereas ACN is only a weak hydrogen bond acceptor. Therefore, the different hydrogen bond interactions involved in the chiral stationary phase, benoxacor enantiomers, and mobile phase resulted in differences in performance between MEOH and ACN. Under reversed-phase HPLC conditions, compared with a relatively high content, a relatively low content of organic solvent generally resulted in an increased retention time and improved separation resolution for enantiomers. The increases in the k_1_, k_2_, and R_s_ values were consistent with this phenomenon when the proportion of ACN or MEOH in the mobile phase decreased. However, the R_s_ increased with increases in MEOH on a Lux Cellulose-1 column, and the maximum R_s_ was obtained at an MEOH/H_2_O ratio of 100/0. Furthermore, the normal-phase HPLC performed better than the reversed-phase HPLC in separating benoxacor enantiomers.

### 2.3. Influence of Temperature on Separation

**Figure 2:**
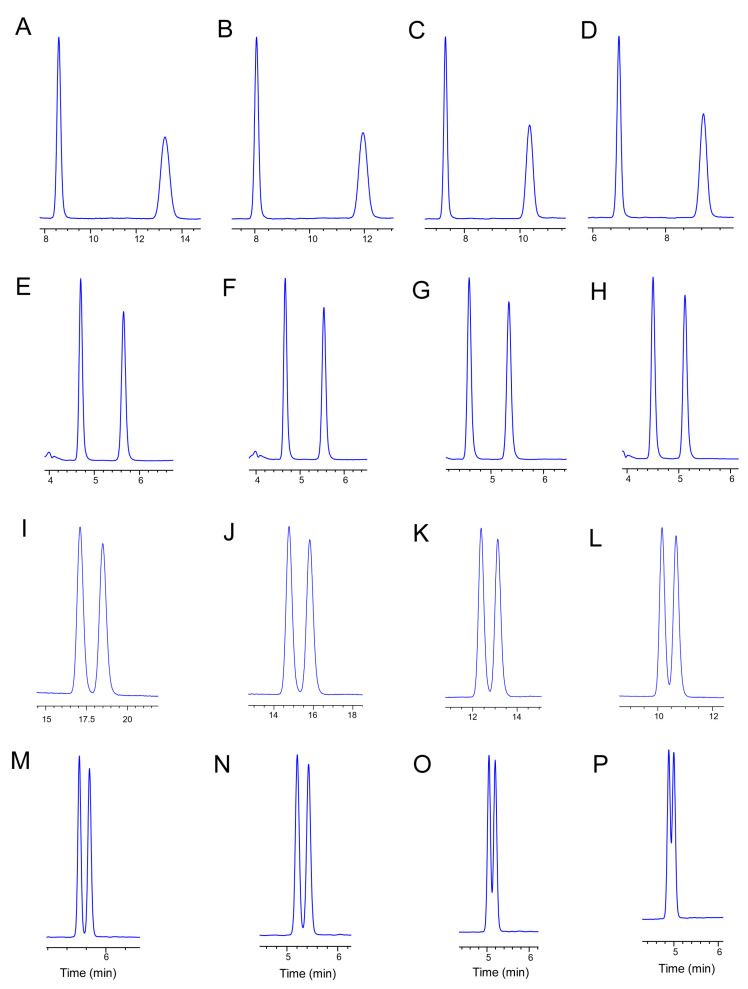
The effects of temperature on benoxacor enantiomer separation with the Lux cellulose-1 (MEOH/H_2_O = 95/5, (**A**) 10 °C, (**B**) 20 °C, (**C**) 30 °C, (**D**) 40 °C), Lux cellulose-3 (ACN/H_2_O = 80/20, (**E**) 10 °C, (**F**) 20 °C, (**G**) 30 °C, (**H**) 40 °C), Lux cellulose-3 (MEOH/H_2_O = 95/5, (**I**) 10 °C, (**J**) 20 °C, (**K**) 30 °C, (**L**) 40 °C), and Chiralpak IC (MEOH/H_2_O = 90/10, (**M**) 10 °C, (**N**) 20 °C, (**O**) 30 °C, (**P**) 40 °C) columns.

Temperature is a key factor in enantiomer separation and helps to unravel the mechanism involved in the chiral separation process. To investigate the influence of temperature on enantiomer separation, the column temperature was increased from 10 °C to 40 °C on the Chiralpak AD, Chiralpak IC, Lux Cellulose-1, Lux Cellulose-3, and Lux Cellulose-2 columns (Figure 2). In the normal-phase condition, considering the retention time and chiral resolution, the separation parameters were HEX/ETOH = 95/5 and HEX/IPA = 95/5 with Chiralpak AD, HEX/ETOH = 95/5 and HEX/IPA = 90/10 with Chiralpak IC, HEX/ETOH = 95/5 and HEX/IPA = 75/25 with Lux Cellulose-1, HEX/ETOH = 85/15 and HEX/IPA = 80/20 with Lux Cellulose-3, and HEX/ETOH = 95/5 with Lux Cellulose-2. In the reversed-phase condition, benoxacor enantiomers were separated on Chiralpak IC (MEOH/H_2_O = 90/10), Lux Cellulose-3 (ACN/H_2_O = 60/40 and MEOH/H_2_O = 90/10), and Lux Cellulose-1 (MEOH/H_2_O = 95/5). The influences of temperature on the separation resolution are summarized in Table S1. Temperature significantly affected benoxacor enantiomer separation under both normal-phase and reversed-phase conditions. Generally, compared with higher temperatures, lower temperatures frequently resulted in higher resolutions, wider peaks, and longer retention times. As a result, the k_1_, k_2_, and R_s_ values of the benoxacor enantiomers decreased as the temperature increased on Lux Cellulose-1 (HEX/IPA = 75/25, HEX/ETOH = 95/5, and MEOH/H_2_O = 95/5), Lux Cellulose-2 (HEX/ETOH = 95/5), Lux Cellulose-3 (HEX/ETOH = 85/15, ACN/H_2_O = 80/20, and MEOH/H_2_O = 95/5), and Chiralpak IC (HEX/IPA = 90/10, HEX/ETOH = 95/5, and MEOH/H2O = 90/10) columns under normal-phase and reversed-phase HPLC conditions. For example, on a Lux Cellulose-1 column with an HEX/ETOH ratio of 95/5 under the normal-phase condition, the k_1_, k_2_, and R_s_ values of the benoxacor enantiomers decreased from 2.29 to 1.60, 2.84 to 1.80, and 2.73 to 1.29, respectively. In some cases, the temperature had little effect on the enantiomer separation. For example, the maximum R_s_ value of 1.79 for the benoxacor enantiomers was obtained at 25 °C on a Chiralpak AD column with a HEX/ETOH ratio of 95/5. 

### 2.4. Thermodynamic Parameters

**Figure 3:**
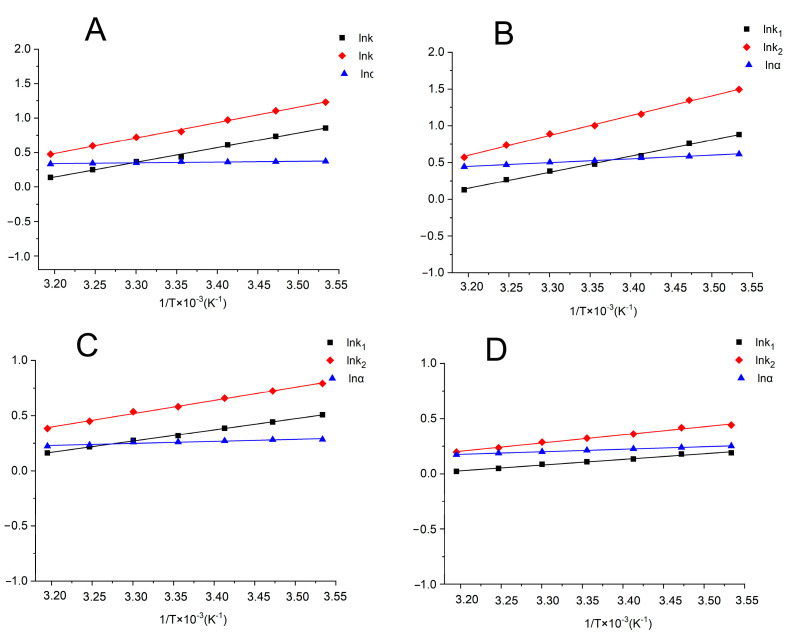
Van’t Hoff plots of benoxacor on Lux Cellulose-3 ((**A**), HEX/ETOH = 85/15; (**B**), HEX/IPA = 80/20) and Chirapak IC column ((**C**), HEX/ETOH = 95/5; and (**D**), HEX/IPA = 90/10).

The Van’t Hoff equation was used to calculate the thermodynamic parameters of ΔH and ΔS based on the retention factor (k) and separation factor (α) obtained from the Chiralpak AD, Chiralpak IC, Lux Cellulose-1, Lux Cellulose-2, and Lux Cellulose-3 columns under different temperatures (Figure 3). Those parameters can reveal the thermodynamic driving forces involved in benoxacor enantiomer separation. Table 2 lists the thermodynamic parameters of benoxacor enantiomers on the five chiral columns under normal-phase and reversed-phase conditions. The linear regressions of lnk versus 1/T and lnα versus 1/T had good linearity, and the ΔH on the Chiralpak AD, Chiralpak IC, Lux Cellulose-1, Lux Cellulose-3, and Lux Cellulose-2 columns ranged from −4.26 KJ/mol to −22.41 KJ/mol under normal-phase HPLC. Similarly, the ΔH values of benoxacor enantiomers on the Chiralpak IC, Lux Cellulose-3, and Lux Cellulose-1 columns ranged from −4.43 KJ/mol to −14.33 KJ/mol under reversed-phase HPLC. The negative values of ΔH showed that under normal-phase and reversed-phase conditions, enthalpy was primarily responsible for the processes of transferring benoxacor enantiomers from the mobile phase to various CSPs. Under both normal-phase and reversed-phase conditions, ΔΔH and ΔΔS ranged from −0.58 KJ/mol to –4.22 KJ/mol and from −0.06 J/mol to −9.77 J/mol, respectively. The negative ΔΔH indicates that the resolution increased at low temperatures. According to previous studies, hydrogen bonding, π–π interactions, and dipole–dipole interactions are the main forces involved in chiral separation [19,29]. The excellent linearity of lnα vs 1/T suggests that it is one of the primary forces involved in benoxacor enantiomer separation. Similarly, the weak linearity suggests that several interaction forces are involved in the separation of benoxacor enantiomers [30,31]. For example, the poor linearity of lnα vs. 1/T on a Chiralpak AD column (R^2^ of 0.667 and 0.339) indicates that multiple interaction forces operate between chiral stationary phases and benoxacor enantiomers in the Chiralpak AD chiral column [31].

### 2.5. Enantiomers Stability and Racemization in Solvents

The stability and racemization of benoxacor enantiomers in MEOH, ETOH, IPA, ACN, HEX, and H_2_O (pH = 4.0, 7.0, and 9.0) were investigated. The benoxacor enantiomers were stable, and no degradation was observed in the six solvents. In addition, the chiral HPLC analysis indicated that no racemization occurred in the solvents. Thus, benoxacor enantiomers were stable, and no racemization occurred in the solvents.

### 2.6. Detection of Benoxacor Enantiomer in Horticultural Soils

According to the elution order reported by Liu [2], the first eluted enantiomer on a Lux Cellulose-3 column is R-benoxacor, and the second is S-benoxacor. The recovery of benoxacor enantiomers in soil ranged from 89.05% to 96.57%, with a coefficient of variation (CV) of less than 5.77% (Table 3). The limit of detection (LOD) was 0.02 mg/L with a signal to noise ratio of 3 (S/N = 3) and the limit of quantitation (LOQ) was 0.05 μg/g for benoxacor enantiomers.

### 2.7. Degradation of Benoxacor Enantiomers in Horticultural Soils

**Figure 4:**
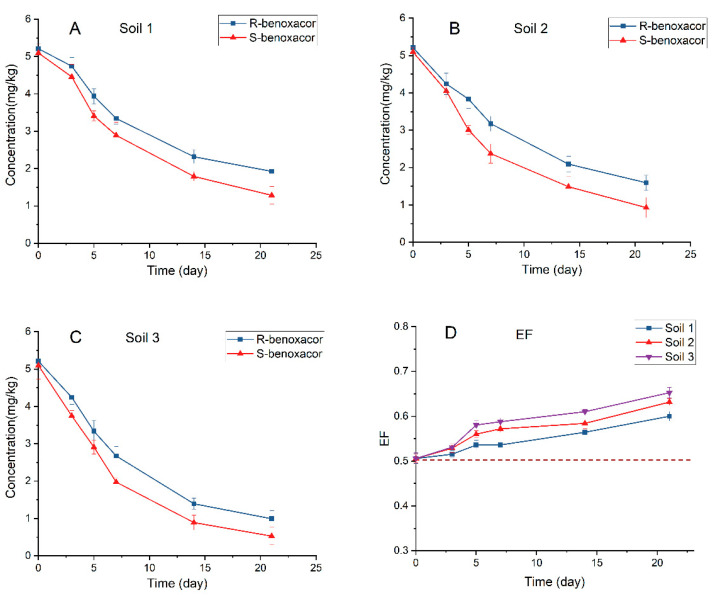
Degradation curves and EF of benoxacor enantiomers in three horticultural soils. (**A**) Soil 1; (**B**) Soil 2; (**C**) Soil 3; (**D**) EF.

The selective degradation of benoxacor enantiomers in three horticultural soils was investigated (Figure 4). The soils were collected from three geographical districts with differences in physicochemical properties, including the pH, organic carbon content, particle size, and soil texture (Table S2). According to the degradation plots of benoxacor enantiomers in soils, after 28 consecutive days of incubation, over 80% of initially spiked benoxacor enantiomers were degraded. The degradation parameters of T_1/2_, k, and *R*^2^ are listed in Table 4. The degradation half-life of R-benoxacor ranged from 8.4 to 13.9 days, with *R*^2^ values from 0.8766 to 0.9715. For S-benoxacor, T_1/2_ ranged from 6.2 to 10.2 days, with *R*^2^ values from 0.9200 to 0.9783. S-benoxacor degraded faster than R-benoxacor in all three horticultural soils, resulting in soil enrichment with R-benoxacor. The T_1/2_ values indicate that benoxacor enantiomers degraded fastest in soil 3 and slowest in soil 1. The EF values increased and were greater than 0.5 with an increased incubation time, suggesting the preferential degradation of S-benoxacor in the three soils.

Enantio-selective degradation of chiral pesticides in soil generally depends heavily on the soil microbial decomposition [32]. Thus, to verify whether the enantio-selective degradation of benoxacor enantiomers in soil is microbe-dependent, the three test soils were sterilized. Figure S1 shows the degradation plots of benoxacor enantiomers in sterilized soils. Less than 20% of initial spiked benoxacor enantiomers were degraded in the three sterilized soils, with degradation rates occurring in the order soil 3 > soil 2 > soil 1 (Figure S1). Moreover, because the concentrations of R-benoxacor and S-benoxacor were nearly the same at all sampling times, the degradation of benoxacor enantiomers in sterilized soils was apparently not enantio-selective. In contrast, over 80% of benoxacor enantiomers degraded in nonsterilized soil after 28 days of incubation. Thus, microbial degradation was significantly more rapid than abiotic degradation in soils and was responsible for the selective breakdown of benoxacor enantiomers in soils. Furthermore, the highest degradation rate of benoxacor enantiomers in sterilized soil 3 might be due to the pH of the soil.

## 3. Materials and Methods

### 3.1. Chemicals and Regents

Racemic benoxacor (Rac-benoxacor; CAS: 98730-04-2; 98% purity) was purchased from Aladdin Reagent Co., Ltd. (Shanghai, China). Benoxacor stock solution was prepared with methanol and stored at 4 °C. The HPLC-grade solvents included ACN, ETOH, MEOH, HEX, and IPA, which were purchased from Honeywell Research Chemicals (Charlotte, NC, USA). Water was generated by a Milli-Q system (Billerica, MA, USA). All other regents were of analytical grade and were purchased commercially.

### 3.2. Apparatus

In the normal-phase chiral HPLC analysis, benoxacor enantiomers were separated with a Shimadzu LC-20A HPLC system (Tokyo, Japan), which included two LC-20AD pumps, a DGU-20A degasser, a CTO-20A column oven, a SIL-20AD sample injector, and an SPD-20A photodiode array detector. Signal collection and data analysis were processed by Shimadzu Labsolution (Tokyo, Japan). In the reversed-phase analysis, an Agilent 1260 series HPLC was used to separate benoxacor enantiomers, which included a G1311B pump, a G1315D diode array detector, a G1329B autosampler, a G1316A column compartment, and a G1322A degasser (Santa Clara, CA, USA). Signals were collected and analyzed using an Agilent Chemstation.

### 3.3. Chiral Columns and Chromatographic Conditions

The Chiralpak AD, Chiralpak IC, Lux Cellulose-1, Lux Cellulose-2, Lux Cellulose-3, and Lux Cellulose-4 columns were used to separate benoxacor enantiomers and were purchased from commercial sources. Benoxacor enantiomers were separated using isocratic elution with HEX/ETOH or HEX/IPA in the normal-phase condition, whereas MEOH/H_2_O and ACN/H_2_O were used in the reversed-phase HPLC condition. In each run, the flow rate of the mobile phase was 0.8 mL/min. For each sample, 10 μL was injected, and the detection wavelength was set at 230 nm. The column temperature was maintained at 20 °C in the mobile phase composition and ratio study and was varied from 10 °C to 40 °C in the thermodynamic study.

### 3.4. Enantiomer Stability and Racemization in Solvents

For an individual benoxacor enantiomer in HEX at 100 mg/L, 100 μL was pipetted into a 2 mL brown sample vial. After the solution had been blown to dryness under nitrogen, 1.0 mL of MEOH, ETOH, IPA, ACN, HEX, or H_2_O (pH = 4.0, 7.0, and 9.0) was added to the vial. Vials were capped, with the caps covered with parafilm, and kept in an incubator in the dark at 25 ± 1 °C. After 0, 6, 12, 24, 48, 72, and 120 h of incubation, triplicate sample vials were removed, dried under nitrogen, and redissolved in 1.0 mL of ACN for reversed-phase chiral HPLC analysis. For samples with water (pH = 4.0, 7.0, and 9.0), vial solutions were directly injected into the HPLC system. All samples were analyzed on the basis of the developed method in separation studies. 

### 3.5. Data Analysis

The separation and thermodynamic parameters, including the separation factor (α), retention factor (*k*), resolution factor (*R_s_*), enthalpy (Δ*H*), and entropy (Δ*S*), were calculated using Equations (1)–(5).

(1) $$k=\frac{\left(t-t_{0}\right)}{t_{0}}$$

(2) $$\alpha =\frac{k_{2}}{k_{1}}$$

(3) $$R_{s}=\frac{2(t_{2}-t_{1})}{w_{1}+w_{2}}$$

(4) $$\ln k=-\frac{\Delta H}{RT}+\frac{\Delta S}{R}+\ln\phi$$

(5) $$\ln\alpha =-\frac{\Delta \Delta H}{RT}+\frac{\Delta \Delta S}{R}$$

In the equations, *t*_0_, *t*_1_, and *t*_2_ are the void time and retention time of benoxacor enantiomers on different chiral columns. The variable w is the peak width of an enantiomer, and ∆∆*S* and ∆∆*H* are the values of ∆*S*_2_ − ∆*S*_1_ and ∆*H*_2_ − ∆*H*_1_, respectively.

The degradation constants (k), half-lives (T_1/2_), and enantiomer fractions (EF) of the benoxacor enantiomers in soils were calculated using Equations (6)–(8).

C_t_ = C_0_e^−kt^ (6)

T_1/2_ = ln2/k (7)

EF = R-benoxacor/(R-benoxacor + S-benoxacor)(8)

In the equations, C_0_ and C_t_ are the benoxacor concentrations in soils at time 0 and time t, respectively. When the EF value deviates from 0.5, enantio-selective degradation of benoxacor in soil is indicated.

### 3.6. Degradation of Benoxacor Enantiomers in Horticultural Soils

Three horticultural soils with different physicochemical properties were used in the benoxacor degradation study. Table S2 shows the locations and properties of the soils, including the particle size, pH, and texture. The initial incubation concentration for Rac-benoxacor was 10 mg/kg (5 mg/kg each for R-benoxacor and S-benoxacor) in dry soil. The water content was adjusted to 36%, and all soil samples were placed in an incubator at 20 ± 1 °C and 60% humidity with a 12 h/12 h light/dark photoperiod for 28 days. Dry soil (5 g) was collected at 0, 1, 3, 5, 7, 14, 21, and 28 days. All samples were stored at −20 °C before analysis. The same procedures were also used to examine the degradation of benoxacor in sterilized soils. 

### 3.7. Sample Preparation

The sample preparation method was optimized based on the procedures reported by Su et al. [1]. To thawed soil sample tubes, 1 g of NaCl, 5 g of Na_2_SO_4_, and 25 mL of ACN were added. Then, samples were shaken for 30 min at 280 rpm, subjected to ultrasonic extraction for 10 min, and then centrifuged at 4000 rpm for 3 min. Extracts were filtered through 10 g of Na_2_SO_4_ for dehydration, and then samples were extracted with ACN a second time. Extracts were evaporated to dryness at 40 °C and resolved in 1.0 mL of MEOH for analysis.

### 3.8. Benoxacor Enantiomer Analysis

Based on the chiral separation results, a Lux Cellulose-3 column was used to analyze benoxacor enantiomers in soil using MEOH/H_2_O (90/10) with a flow rate of 0.8 mL/min. The accuracy of the analytical method was evaluated by the recovery, whereby the actually detected concentrations were compared to the spiked concentrations in blank soil at different levels. The precision of the analytical method was evaluated by the coefficient of variation (CV). The limit of detection (LOD) was defined as the concentration that produced a signal-to-noise (S/N) ratio of 3, and the limit of quantitation (LOQ) was the lowest concentration in the method accuracy experiments.

## 4. Conclusions

In this study, the separation of benoxacor enantiomers on six commercial chiral columns was investigated by HPLC under normal-phase and reversed-phase conditions. Under normal-phase conditions, the two benoxacor enantiomers were completely separated on the Chiralpak AD, Chiralpak IC, Lux Cellulose-1, and Lux Cellulose-3 columns and partially separated on a Lux Cellulose-2 column. Under reversed-phase conditions, the benoxacor enantiomers were completely separated on a Lux Cellulose-3 column and partially separated on the Chiralpak IC and Lux Cellulose-1 columns. Normal-phase HPLC performed better than reversed-phase HPLC in terms of benoxacor enantiomer separation, and Lux Cellulose-3 performed the best under normal-phase and reverse-phase HPLC. The benoxacor enantiomers were stable, and no degradation or racemization was observed in MEOH, ETOH, IPA, ACN, HEX, or H_2_O (pH = 4.0, 7.0, and 9.0). Furthermore, S-benoxacor degraded faster than R-benoxacor in the three horticultural soils, and the enantio-selective degradation rates of benoxacor in soil were highly related to the pH and soil microbial activity. The results of this study will help to improve the risk assessment of enantiomer levels of benoxacor in the environment.

## Figures and Tables

**Figure 1:**
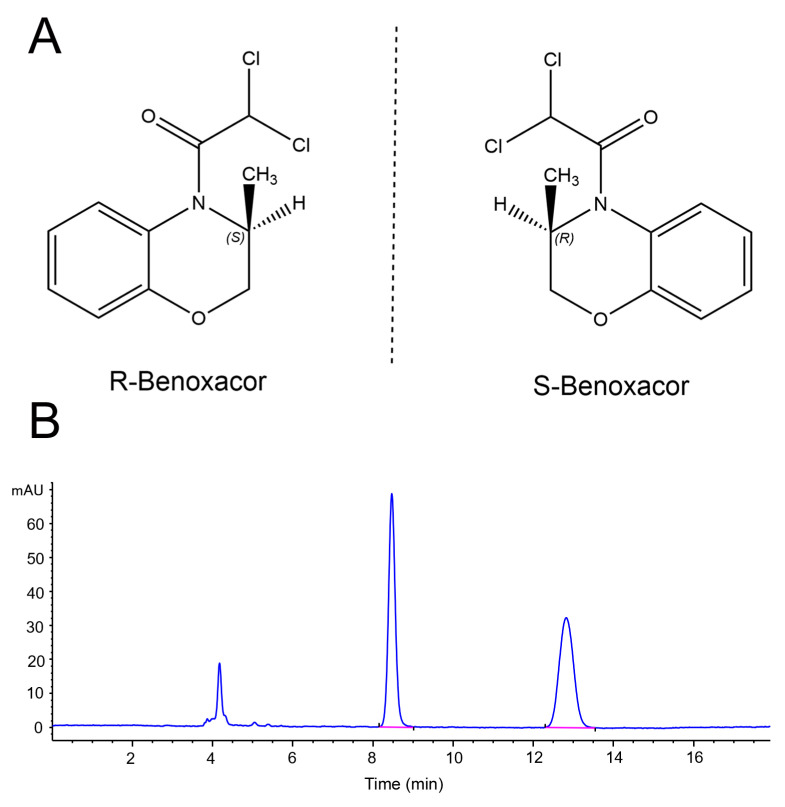
Chemical structure (**A**) and representative chromatogram (**B**) of benoxacor enantiomers.

**Table 1:** Enantiomeric separation of benoxacor enantiomers on six chiral columns.

Column	Mobile Phase	Ratio (*v*/*v*)	α	Rs	Mobile Phase	Ratio (*v*/*v*)	α	Rs
Lux Cellulose-1	HEX/IPA	95/5	1.02	0.38	MEOH/H_2_O	100/0	1.13	0.54
		90/10	1.04	0.50		95/5	1.09	0.53
		85/15	1.04	0.63		90/10	1.07	0.52
		80/20	1.05	0.70		85/15	1.06	0.47
		75/25	1.06	0.71		80/20	1.04	0.43
	HEX/ETOH	98/2	1.21	2.57	ACN/H_2_O	/	/	/
		95/5	1.17	2.22		/	/	/
		90/10	1.13	1.48		/	/	/
		85/15	1.11	1.00		/	/	/
		80/20	1.09	0.72		/	/	/
		75/25	1.08	0.72		/	/	/
Lux Cellulose-2	HEX/IPA	/	/	/	MEOH/H_2_O	/	/	/
	HEX/ETOH	98/2	1.04	0.68	ACN/H_2_O	/	/	/
		95/5	1.06	0.71		/	/	/
		90/10	1.06	0.66		/	/	/
Lux Cellulose-3	HEX/IPA	95/5	1.49	4.15	MEOH/H_2_O	95/5	1.96	2.92
		90/10	1.47	3.30		90/10	2.08	3.91
		85/15	1.46	3.38		85/15	2.18	5.13
		80/20	1.45	2.74		80/20	2.18	6.08
		75/25	1.43	2.53		/	/	/
	HEX/ETOH	98/2	1.52	4.69	ACN/H_2_O	90/10	2.40	0.95
		95/5	1.50	3.42		80/20	2.29	1.46
		90/10	1.69	5.81		70/30	2.19	2.19
		85/15	1.69	6.16		60/40	2.12	3.26
		80/20	1.70	5.64		50/50	2.51	5.17
		75/25	1.68	4.86		/	/	/
Lux Cellulose-4	HEX/IPA	/	/	/	MEOH/H_2_O	/	/	/
	HEX/ETOH	/	/	/	ACN/H_2_O	/	/	/
Chiralpak AD	HEX/IPA	98/2	1.09	1.14	MEOH/H_2_O	/	/	/
		95/5	1.09	0.98		/	/	/
		90/10	1.09	0.88		/	/	/
		85/15	1.09	0.84		/	/	/
		80/20	1.09	0.81		/	/	/
		75/25	1.09	0.79		/	/	/
	HEX/ETOH	98/2	1.35	3.52	ACN/H_2_O	/	/	/
		95/5	1.18	1.79		/	/	/
		90/10	1.11	1.23		/	/	/
		85/15	1.07	0.81		/	/	/
		80/20	1.04	0.53		/	/	/
		75/25	1.03	0.34		/	/	/
Chiralpak IC	HEX/IPA	98/2	1.28	3.45	MEOH/H_2_O	90/10	1.15	0.57
		95/5	1.28	2.38		85/15	1.14	0.69
		90/10	1.30	2.66		80/20	1.13	0.74
		85/15	1.29	2.17		75/25	1.12	0.84
		80/20	1.29	1.83		70/30	1.11	0.96
		75/25	1.29	1.79		/	/	/
	HEX/ETOH	98/2	1.24	2.50	ACN/H_2_O	/	/	/
		95/5	1.27	2.97		/	/	/
		90/10	1.24	1.50		/	/	/
		85/15	1.21	1.28		/	/	/
		80/20	1.21	1.37		/	/	/
		75/25	1.19	1.04		/	/	/

**Table 2:** Van’t Hoff equations and thermodynamic parameters of benoxacor enantiomers with five chiral columns.

Column	Mobile Phase	Lnk = −△H/RT + △S/R + lnφ	R^2^	△H (KJ mol^−1^)	△S/R + lnφ	lnα = −∆∆H/RT + ∆∆S/R	R^2^	△△H (KJ mol^−1^)	△△S (J mol^−1^)
Lux Cellulose-1	MEOH/H_2_O (95/5)	y = 750.98x − 3.5623	0.982	−6.24	−3.56	y = 218.66x − 0.6079	0.961	−1.82	−5.05
		y = 969.63x − 4.1702	0.980	−8.06	−4.17				
Lux Cellulose-1	HEX/IPA (75/25)	y = 1347x − 4.4026	0.994	−11.20	−4.40	y = 124.7x − 0.358	0.959	−1.04	−2.98
		y = 1471.7x − 4.7606	0.995	−12.24	−4.76				
Lux Cellulose11	HEX/ETOH (95/5)	y = 1088.3x − 3.0047	0.993	−9.05	−3.00	y = 312.42x − 0.8844	0.994	−2.60	−7.35
		y = 1400.8x − 3.8891	0.993	−11.65	−3.89				
Lux Cellulose-2	HEX/ETOH (95/5)	y = 849.05x − 2.4806	0.985	−7.06	−2.48	y = 69.467x − 0.1735	0.958	−0.58	−1.44
		y = 918.52x − 2.654	0.984	−7.64	−2.65				
Lux Cellulose-3	MEOH/H_2_O (90/10)	y = 1456x − 5.0499	0.982	−12.11	−5.05	y = 268.1x − 0.2293	0.987	−2.23	−1.91
		y = 1724.1x − 5.2792	0.985	−14.33	−5.28				
Lux Cellulose-3	ACN/H_2_O (60/40)	y = 532.77x − 3.5984	0.968	−4.43	−3.60	y = 412.04x − 0.5817	0.952	−3.43	−4.84
		y = 944.82x − 4.1801	0.976	−7.86	−4.18				
Lux Cellulose-3	HEX/IPA (80/20)	y = 2126.3x − 6.6577	0.995	−17.68	−6.66	y = 108.67x − 0.007	0.866	−0.90	−0.06
		y = 2235x − 6.6647	0.997	−18.58	−6.66				
Lux Cellulose-3	HEX/ETOH (85/15)	y = 2188x − 6.852	0.996	−18.19	−6.85	y = 507.3x − 1.1748	0.995	−4.22	−9.77
		y = 2695.3x − 8.0267	0.998	−22.41	−8.03				
Lux Amylose-1	HEX/IPA (95/5)	y = 1210x − 2.9493	0.997	−10.06	−2.95	y = −87.024x + 0.3683	0.667	0.72	3.06
		y = 1122.9x − 2.581	0.990	−9.34	−2.58				
Lux Amylose-1	HEX/ETOH (95/5)	y = 1316.1x − 3.538	0.977	−10.94	−3.54	y = −27.113x + 0.3046	0.339	0.23	2.53
		y = 1289x − 3.2334	0.984	−10.72	−3.23				
Chirapak IC	MEOH/H_2_O (90/10)	y = 1110.5x − 3.5073	0.985	−9.23	−3.51	y = 96.821x − 0.2132	0.993	−0.80	−1.77
		y = 1207.4x − 3.7205	0.987	−10.04	−3.72				
Chirapak IC	HEX/IPA (90/10)	y = 1015x − 3.0798	0.999	−8.44	−3.08	y = 182.81x − 0.3551	0.916	−1.52	−2.95
		y = 1197.8x − 3.4348	0.997	−9.96	−3.43				
Chirapak IC	HEX/ETOH (95/5)	y = 511.9x − 1.6097	0.988	−4.26	−1.61	y = 228.8x − 0.5555	0.999	−1.90	−4.62
		y = 740.7x − 2.1653	0.994	−6.16	−2.17				

**Table 3:** Precision and accuracy of the analytical method for benoxacor enantiomers in the soil at three spiked levels.

Compound	Spiked Level (μg/g)	Intraday						Interday
		Day 1		Day 2		Day 3		
		Recovery (%)	CV ^a^ (%)	Recovery (%)	CV ^a^ (%)	Recovery (%)	CV ^a^ (%)	CV ^b^ (%)
R-benoxacor	0.05	91.78	1.47	93.66	3.78	94.78	1.63	2.86
	0.5	91.48	5.03	92.58	4.70	92.31	4.25	4.70
	5	96.57	2.85	94.46	1.89	96.16	5.05	3.66
S-benoxacor	0.05	91.15	3.71	91.99	4.80	90.12	1.95	3.79
	0.5	93.50	3.41	89.05	2.03	92.25	5.24	4.34
	5	92.18	5.61	93.01	5.77	93.88	4.00	5.23

^a^ Intraday CV (n = 5). ^b^ Interday CV (n = 15).

**Table 4:** First-order rate constants (k), half-lives (T_1/2_), and correlation coefficient (R^2^) values of benoxacor degradation in soils.

Soil No.	Site	BENO Enantiomer	k (day^−1^)	t_1/2_ (day)	R^2^
Soil 1	Guangxi	R-benoxacor	0.0500	13.9	0.8766
		S-benoxacor	0.0678	10.2	0.9321
Soil 2	Chongqing	R-benoxacor	0.0574	12.1	0.9114
		S-benoxacor	0.0809	8.6	0.9200
Soil 3	Gansu	R-benoxacor	0.0825	8.4	0.9715
		S-benoxacor	0.1115	6.2	0.9783

## Data Availability

The data are available on request to the corresponding author.

## Supplementary Materials

The following supporting information can be downloaded at: https://www.mdpi.com/article/10.3390/ijms24108887/s1.
