# Supplementary material for: Enantiomeric Separation and Degradation of Benoxacor Enantiomers in Horticultural Soil by Normal-Phase and Reversed-Phase High Performance Liquid Chromatography
Source: Int J Mol Sci. 2023 May 17;24(10):8887. doi: 10.3390/ijms24108887 (PMC10218933; doi:10.3390/ijms24108887)
Supplement: Supplementary file 1 [file ijms-24-08887-s001.zip › ijms-2385639-supplementary.pdf]

# Supplementary Materials

## Enantiomeric Separation and Degradation of Benoxacor Enantiomers in Horticultural Soil by Normal-phase and Reversed-phase High Performance Liquid Chromatography

**Haoxiang Zhu[1], Jinhui Cheng[2], Kunrong Qin[1], Ping Zhang[2,*] and Haiyang Wang[1,*]**

[1] College of Horticulture and Landscape Architecture, Southwest University, Chongqing 400715, China
[2] College of Plant Protection, Southwest University, Chongqing 400715, China
* Correspondence: pingz028@163.com (P.Z.) whyswau@126.com (H.W.); Tel.: +86-23-68250541; Fax.: +86-23-68251274

**Table S1.** Effects of temperature on benoxacor enantiomers separation with five chiral columns

| Stationary phase | Mobile phase (v/v) | Tep | k1 | k2 | α | Rs | Mobile phase (v/v) | Tep | k1 | k2 | α | Rs |
|---|---|---|---|---|---|---|---|---|---|---|---|---|
| Lux Cellulose-1 | HEX/IPA(75/25) | 10 | 1.41 | 1.53 | 1.08 | 0.84 | MEOH/$H_2O$(95/5) | 10 | 0.40 | 0.46 | 1.17 | 0.59 |
| | | 15 | 1.33 | 1.43 | 1.08 | 0.77 | | 15 | 0.39 | 0.46 | 1.17 | 0.61 |
| | | 20 | 1.24 | 1.33 | 1.07 | 0.67 | | 20 | 0.37 | 0.43 | 1.16 | 0.55 |
| | | 25 | 1.11 | 1.18 | 1.06 | 0.69 | | 25 | 0.35 | 0.40 | 1.13 | 0.51 |
| | | 30 | 1.03 | 1.09 | 1.06 | 0.56 | | 30 | 0.34 | 0.38 | 1.12 | 0.52 |
| | | 35 | 0.97 | 1.01 | 1.04 | 0.49 | | 35 | 0.32 | 0.36 | 1.11 | 0.38 |
| | | 40 | 0.91 | 0.95 | 1.04 | 0.38 | | 40 | 0.31 | 0.34 | 1.10 | 0.34 |
| | HEX/ETOH(95/5) | 10 | 2.29 | 2.84 | 1.24 | 2.73 | ACN/$H_2O$(80/20) | 10 | / | / | / | / |
| | | 15 | 2.21 | 2.72 | 1.23 | 2.87 | | 15 | / | / | / | / |
| | | 20 | 2.04 | 2.44 | 1.20 | 2.31 | | 20 | / | / | / | / |
| | | 25 | 1.90 | 2.24 | 1.18 | 2.22 | | 25 | / | / | / | / |
| | | 30 | 1.78 | 2.06 | 1.16 | 1.88 | | 30 | / | / | / | / |
| | | 35 | 1.71 | 1.95 | 1.14 | 1.51 | | 35 | / | / | / | / |
| | | 40 | 1.60 | 1.80 | 1.12 | 1.29 | | 40 | / | / | / | / |
| Lux Cellulose-2 | HEX/ETOH(95/5) | 10 | 1.70 | 1.83 | 1.08 | 0.90 | ACN/$H_2O$ | 10 | / | / | / | / |
| | | 15 | 1.60 | 1.71 | 1.07 | 0.83 | | 15 | / | / | / | / |
| | | 20 | 1.52 | 1.61 | 1.06 | 0.76 | | 20 | / | / | / | / |
| | | 25 | 1.44 | 1.52 | 1.06 | 0.76 | | 25 | / | / | / | / |
| | | 30 | 1.37 | 1.44 | 1.06 | 0.73 | | 30 | / | / | / | / |
| | | 35 | 1.30 | 1.37 | 1.05 | 0.69 | | 35 | / | / | / | / |
| | | 40 | 1.29 | 1.36 | 1.05 | 0.73 | | 40 | / | / | / | / |
| Lux Cellulose-3 | HEX/IPA(80/20) | 10 | 2.35 | 3.42 | 1.45 | 2.38 | MEOH/$H_2O$(95/5) | 10 | 1.06 | 2.16 | 2.04 | 4.27 |
| | | 15 | 2.09 | 3.02 | 1.44 | 2.31 | | 15 | 1.03 | 2.07 | 2.01 | 4.13 |
| | | 20 | 1.84 | 2.64 | 1.44 | 2.93 | | 20 | 0.94 | 1.88 | 1.99 | 3.79 |
| | | 25 | 1.55 | 2.24 | 1.45 | 2.74 | | 25 | 0.85 | 1.68 | 1.96 | 3.51 |
| | | 30 | 1.45 | 2.06 | 1.42 | 2.71 | | 30 | 0.79 | 1.53 | 1.93 | 3.48 |
| | | 35 | 1.29 | 1.82 | 1.41 | 2.25 | | 35 | 0.71 | 1.35 | 1.90 | 3.18 |
| | | 40 | 1.15 | 1.61 | 1.40 | 2.28 | | 40 | 0.67 | 1.24 | 1.87 | 2.93 |
| | HEX/ETOH(85/15) | 10 | 2.41 | 4.46 | 1.85 | 6.35 | ACN/$H_2O$(80/20) | 10 | 0.18 | 0.42 | 2.36 | 1.60 |
| | | 15 | 2.14 | 3.84 | 1.80 | 6.37 | | 15 | 0.18 | 0.41 | 2.35 | 1.56 |
| | | 20 | 1.81 | 3.18 | 1.76 | 5.35 | | 20 | 0.17 | 0.39 | 2.29 | 1.54 |
| | | 25 | 1.61 | 2.72 | 1.69 | 6.16 | | 25 | 0.16 | 0.37 | 2.25 | 1.46 |
| | | 30 | 1.47 | 2.43 | 1.66 | 5.25 | | 30 | 0.16 | 0.35 | 2.21 | 1.44 |
| | | 35 | 1.31 | 2.09 | 1.60 | 5.10 | | 35 | 0.15 | 0.33 | 2.13 | 1.32 |
| | | 40 | 1.14 | 1.77 | 1.56 | 4.96 | | 40 | 0.15 | 0.31 | 2.05 | 1.16 |
| Chiralpak AD | HEX/IPA(95/5) | 10 | 3.72 | 3.92 | 1.05 | 0.95 | MEOH/$H_2O$ | 10 | / | / | / | / |
| | | 15 | 3.52 | 3.75 | 1.07 | 0.99 | | 15 | / | / | / | / |
| | | 20 | 3.27 | 3.57 | 1.09 | 0.97 | | 20 | / | / | / | / |
| | | 25 | 3.05 | 3.29 | 1.08 | 1.14 | | 25 | / | / | / | / |
| | | 30 | 2.85 | 3.09 | 1.08 | 1.06 | | 30 | / | / | / | / |
| | | 35 | 2.68 | 2.91 | 1.09 | 0.99 | | 35 | / | / | / | / |
| | | 40 | 2.47 | 2.70 | 1.09 | 0.94 | | 40 | / | / | / | / |

| | | | | | | | | | | | | |
|---|---|---|---|---|---|---|---|---|---|---|---|---|
| | HEX/ETOH(95/5) | 10 | 2.92 | 3.63 | 1.24 | 0.84 | ACN/$H_2O$ | 10 | / | / | / | / |
| | | 15 | 2.89 | 3.55 | 1.23 | 0.80 | | 15 | / | / | / | / |
| | | 20 | 2.67 | 3.29 | 1.23 | 0.89 | | 20 | / | / | / | / |
| | | 25 | 2.41 | 2.99 | 1.24 | 1.79 | | 25 | / | / | / | / |
| | | 30 | 2.22 | 2.75 | 1.24 | 0.93 | | 30 | / | / | / | / |
| | | 35 | 2.08 | 2.58 | 1.24 | 1.28 | | 35 | / | / | / | / |
| | | 40 | 1.94 | 2.41 | 1.24 | 1.48 | | 40 | / | / | / | / |
| Chiralpak IC | HEX/IPA(90/10) | 10 | 1.66 | 2.21 | 1.33 | 2.86 | MEOH/$H_2O$(90/10) | 10 | 1.50 | 1.71 | 1.14 | 0.94 |
| | | 15 | 1.56 | 2.06 | 1.33 | 2.67 | | 15 | 1.40 | 1.59 | 1.13 | 0.93 |
| | | 20 | 1.47 | 1.93 | 1.31 | 2.61 | | 20 | 1.33 | 1.49 | 1.12 | 0.92 |
| | | 25 | 1.38 | 1.79 | 1.30 | 2.66 | | 25 | 1.28 | 1.43 | 1.12 | 0.82 |
| | | 30 | 1.32 | 1.71 | 1.30 | 2.63 | | 30 | 1.19 | 1.32 | 1.11 | 0.81 |
| | | 35 | 1.24 | 1.57 | 1.26 | 2.22 | | 35 | 1.11 | 1.23 | 1.11 | 0.76 |
| | | 40 | 1.18 | 1.47 | 1.25 | 1.85 | | 40 | 1.02 | 1.12 | 1.10 | 0.69 |
| | HEX/ETOH(95/5) | 10 | 1.21 | 1.56 | 1.29 | 2.85 | ACN/$H_2O$ | 10 | / | / | / | / |
| | | 15 | 1.20 | 1.52 | 1.27 | 3.13 | | 15 | / | / | / | / |
| | | 20 | 1.14 | 1.43 | 1.25 | 2.17 | | 20 | / | / | / | / |
| | | 25 | 1.12 | 1.38 | 1.24 | 2.97 | | 25 | / | / | / | / |
| | | 30 | 1.09 | 1.33 | 1.22 | 2.35 | | 30 | / | / | / | / |
| | | 35 | 1.05 | 1.27 | 1.21 | 1.53 | | 35 | / | / | / | / |
| | | 40 | 1.02 | 1.22 | 1.19 | 1.87 | | 40 | / | / | / | / |

**Table S2**. Physicochemical properties of the soils

| Soil No. | site | Particle size | | | | pH | $C_{org}$(%) |
|---|---|---|---|---|---|---|---|
| | | sand | slit | clay | texture | | |
| Soil 1 | Guangxi | 35.7 | 44.3 | 20.0 | slit loam | 5.41 | 2.2 |
| Soil 2 | Chongqing | 23.7 | 17.4 | 58.9 | clay loam | 6.68 | 4.8 |
| Soil 3 | Gansu | 69.1 | 14.6 | 16.3 | sandy loam | 7.94 | 1.9 |

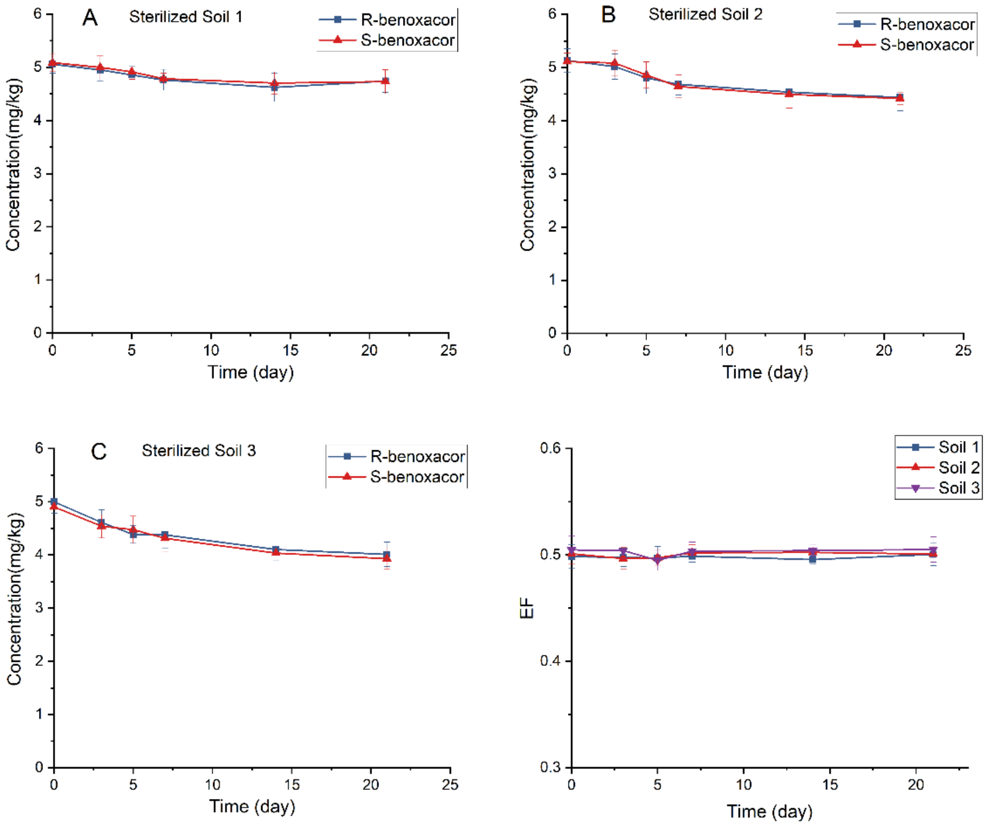


**Figure S1**. Degradation curves of benoxacor enantiomers in three sterilized soils. (**A**) Soil 1; (**B**) Soil 2; (**C**) Soil 3; (**D**) EF.
